# Association of dietary patterns with the newly diagnosed diabetes mellitus and central obesity: a community based cross-sectional study

**DOI:** 10.1038/s41387-020-0120-y

**Published:** 2020-06-04

**Authors:** Xueyao Yin, Yixin Chen, Weina Lu, Ting Jin, Lin LI

**Affiliations:** Department of Endocrinology, the Affiliated Sir Run Run Shaw Hospital, School of Medicine, Zhejiang University, 3 East Qingchun Road, 310016 Hangzhou, China

**Keywords:** Nutrition, Type 2 diabetes

## Abstract

**Aim:**

The purpose of this study was to investigate the association of dietary patterns with the risk of insulin resistance (IR), diabetes mellitus (DM), and central obesity in China.

**Methods:**

We performed a cross-sectional study on 1432 participants, aged 40–65 years in Hangzhou, Zhejiang province, China. Dietary intake was assessed using a semi-quantitative food frequency questionnaire.

**Results:**

Factor analysis extracted four major dietary patterns: vegetable-fruits, rice–meat, seafood–eggs, and sweet–fast. The vegetable-fruits pattern was inversely associated with HOMA-IR (*p* < 0.001 in both genders), while sweet–fast food pattern was significantly associated with higher HOMA-IR (*p* = 0.002 in male, and *p* < 0.001 in female). The vegetables–fruits pattern was inversely correlated with visceral fat area (VFA) (*p* = 0.029 in males, and *p* = 0.017 in females), while sweet–fast food pattern presented a significant direct association (*p* < 0.001 in male) with VFA in males. There was no association observed between the rice–meat pattern or the seafood–eggs pattern and HOMA-IR or VFA. After adjustment for potential confounding factors, participants in the highest tertile of vegetable-fruits pattern showed a significantly lower risk of DM in both males and females (OR: 0.30, 95% CI: 0.13–0.70 in male, and OR: 0.28, 95% CI: 0.11–0.72 in female), and lower risk of central obesity was observed in males (OR: 0.50, 95% CI: 0.29–0.86 in male). Conversely, participants in the highest tertile of sweet–fast food pattern had higher risk of DM (OR: 2.58, 95% CI: 1.23–5.88 in male), and central obesity (OR: 2.85, 95% CI: 1.67–4.86 in male) only in male. While neither the rice–meat pattern nor the seafood–eggs pattern showed significant association with DM or central obesity in both genders.

**Conclusions:**

Our findings indicated low risk of IR, DM, and central obesity with vegetable-fruits pattern while inverse relation with sweet–fast food pattern.

## Introduction

Diabetes mellitus (DM) has become a major public health concern worldwide due to its growing epidemic prevalence. In China, the prevalence of DM dramatically increased among adults from 0.67% in 1980 to 10.9% in 2013^1–6^. There are two major subtypes of DM: type 1 and type 2. Type 2 diabetes (T2DM) is the most common type of DM, affecting 90% of people with diabetes, and is mainly linked to insulin resistance (IR) and relative insufficiency of insulin secretion. The probability of DM was higher in overweight and obese individuals than individuals with normal body mass index (BMI), and was even higher in participants with central obesity^7^. It is well known that obesity has a multifactorial etiology which includes genetic, environmental, and dietary factors. Of these, dietary factors are thought to play a key role.

Approximately 90% of T2DM occurring is attributed to excess weight^8^. Previous studies have reported that body composition, especially body fat, is closely related to glucose metabolism in humans^9,10^. Compared with subcutaneous adipose tissue, visceral adipose tissue is known to release more proinflammatory and proatherogenic factors, leading to exacerbation of oxidative stress and IR. Studies have demonstrated that magnetic resonance imaging (MRI) and computed tomography (CT) are the reference methods to measure the accumulation of visceral fat^11^. Dietary patterns, especially western dietary pattern, have been shown to increase weight and body fat percentage^12,13^. However, few reports have demonstrated the association between dietary patterns and body fat distribution.

Traditional approach that investigates diet and disease associations mainly focuses on a single nutrient or food. However, no one consumes just one single nutrient or food, making it more difficult to separate the effect of individual dietary components. The effect of different combinatorial foods on the body can be studied by dietary pattern analyses. Cumulative studies have revealed that vegetables and fruits^14^, low-fat dairy products^15^, and regular alcohol consumption^16,17^ might reduce the risk of IR. In contrast, the western dietary pattern defined by high energy and fat, and low dietary fiber intake increased the risk of glucose tolerance abnormalities^18–20^.

Due to the rapid social and economic development, dietary patterns, and lifestyles have changed substantially in China. Therefore, the aim of the study was to identify the major Chinese dietary patterns of individuals aged 40–65 years and evaluate their association with body fat distribution, and DM.

## Research design and methods

### Study design

During the period of March to May 2010, a population-based cross-sectional survey was carried out in the Caihe communities of Hangzhou, Zhejiang province, China. Totally 1432 Han Chinese participants aged 40–65 years were included in the present study. Subjects who had a previous history of stroke or ischemic heart disease, and who were previously diagnosed with DM were excluded. The study was conducted in accordance with the Declaration of Helsinki, and all procedures were approved by the ethics committee of Sir Run Run Shaw Hospital. All participants signed an informed consent before data collection.

### Demographic characteristics

Demographic and lifestyle covariates, such as smoking status, alcohol intake, and physical activity were collected by a questionnaire. We identified three categories for smoking: current smoker, former smoker, and non- smoker according to the participants’ cigarette use answer. A participant was defined as “drinker” in case of having drunk alcoholic beverage once per week on average during the last 4 weeks, excluding those who drank alcoholic beverage only during festivals.

### Dietary assessment

A validated semi-quantitative food frequency questionnaire (FFQ) with 81 items was used to assess the food and nutritional intakes by trained dietitians during a structured interview^21,22^. Participants were asked to recall their frequency of intakes of each food item in the previous 4 weeks. The frequency of food intake was measured using seven categories which were as follows: (1) never; (2) <1 time/ week; (3) 1 time/ week; (4) 2–3 times/ week; (5) 4–6 times/ week; (6) 1 time/day; (7) ≥2 times/day. Eighty-one food items were gathered in 21 predefined food groups on the basis of similarity in nutrient profiles, culinary use among the foods and the grouping scheme used in other studies. The amount of each food item consumed on average was calculated by multiplying the frequency of intake by portion size of the food, using China Food Composition Table (2009) as the database.

### Laboratory measurements

Participants were requested not to eat after 20:00 pm, and received a medical checkup from 7:00 to 12:00 am the next day. Venous blood samples were obtained at 0 and 2 hours after a 75 g glucose load for an oral glucose tolerance test (OGTT). Serum glucose concentrations, triglyceride (TG), total cholesterol (TC), high density lipoprotein–cholesterol (HDL-c), and low density lipoprotein–cholesterol (LDL-c) were determined by an autoanalyzer (Aeroset, Chicago, IL, USA). Glycosylated hemoglobin A_1_c (HbA_1_c) in the whole blood was measured by high-performance liquid chromatography (Hemglobin Testing System; Bio-Rad, Hercules, CA, USA). Insulin in the serum was measured by radioimmunology using an insulin detection kit (Beijing North Institute of Biological Technology, China). Homeostasis model of insulin resistance (HOMA-IR) sore was calculated using the following equation: [fasting serum insulin (FINS; mU/L) × fasting serum glucose (FPG; mmol/L)/22.5]^23^.

### Anthropometric measurement

BMI was defined as body weight divided by height squared (kg/m^2^). Waist circumference (WC) was measured at a level halfway between the lower rib cage and iliac crest. Hip circumference was measured at the maximal horizontal girth between the waist and thigh, and the waist–to-hip ratio (WHR) was calculated and recorded for each participant. Both measurements were performed while the participant was standing. Body fat percentage (Fat%) was determined with a bioelectrical impedance analysis system (TBF-300, Tanita Co, Tokyo, Japan). Blood pressure was calculated as the average of three readings, using a mercury sphygmomanometer.

Abdominal adipose tissue was measured using a whole-body imaging system (SMT-100, Shimadzu Co, Kyoto, Japan). Spinecho sequences, TR-500 and TE-200, with a matrix size of 256 × 256 were performed for all participants. Visceral fat area (VFA) and subcutaneous adipose tissue area (SFA) were determined at the abdominal level between L4 and L5 vertebral disc space in the supine position with MRI, which were calculated with the workstation provided by the manufacturer.

### Diagnosis of diabetes and central obesity

DM was diagnosed according to the World Health Organization criteria. Subjects without a history of DM were administered with a 75 g OGTT. We coded participants as DM when they had glucose level above the diagnostic threshold, which is fasting glucose level≥7 mmol/L, and or 2-h glucose level ≥11.1 mmol/L. And VFA ≥ 80 cm^2^ was defined as central obesity^24^.

### Statistical analysis

Sample characteristics were presented as mean ± standard deviation (SD) for normally distributed variables and frequency and percentage for categorical variables. Glucose, insulin, HOMA-IR, TG, VFA, SFA, and total energy intake were reported as median (range) due to skewness and transformed lg(x) before analysis. Sweet–fast food pattern score underwent lg(x + 10) transformation to achieve a normal distribution. To compare the general characteristics according to DM status in patients and controls, continuous variables were tested by student’s-test and Pearson’s χ^2^ test for categorical variables. We used the factor analysis (principal component) to extract the participants’ dietary patterns among the 21 predefined food groups. To improve interpretability, the factors were rotated with an orthogonal rotation (varimax rotation) to minimize the correlation between factors. Four patterns were selected after evaluating the eigenvalue (≥1.5), scree plot, and factor interpretability. Items were remained in a factor if they had an absolute correlation of >±0.25 with that pattern. Correlation analysis and linear regression analysis were used to investigate the association between each dietary pattern score and HOMA-IR, as well as VFA. Factor scores for each pattern were divided into tertiles for further analysis. Multivariate logistic regression analysis was used to examine the relationships between dietary pattern with DM status and central obesity.

All analyses were performed by use of SPSS software (version 16.0 for Windows, SPSS Inc., Chicago, IL, USA). Statistical significance was considered when two-sided *p*-values < 0.05.

## Results

### Subject characteristics

The subject characteristics are presented in Table 1. A total of 575 males and 857 females were surveyed. The prevalence of newly diagnosed DM was observed to be 10.6% in males and 6.1% in females, respectively. There were no significant differences in education, family income, smoking, drinking, physical activity, BMI, systolic blood pressure (SBP), diastolic blood pressure (DBP), 2h insulin (2h INS), TC, LDL-c, SFA, total energy intake between participants with and without DM in both males and females. WC, WHR, Fat%, FPG, 2h postprandia glucose (2h PG), FINS, HOMA-IR, HbA1_C_, HDL-c, VFA were significantly higher in the DM than control group. Compared with participants who did not have DM, those who had DM tended to be older and with higher TG in females, but not in males.

**Table 1:** General characteristics of study population.

Characteristics	Male			Female		
	DM status		*p*	DM status		*p*
	No (514, 89.4%)	Yes (61, 10.6%)		No (805, 93.9%)	Yes (52. 6.1%)	
Age (year)	56.88 ± 7.53	58.82 ± 5.46	0.065	56.86 ± 7.59	60.47 ± 6.90	0.001
BMI (kg/m^2^)	23.88 ± 2.89	24.47 ± 2.76	0.159	23.12 ± 2.91	23.59 ± 3.38	0.286
WC (cm)	82.75 ± 8.70	85.34 ± 7.67	0.039	75.40 ± 8.04	78.37 ± 9.25	0.015
WHR	0.91 ± 0.06	0.93 ± 0.05	0.013	0.84 ± 0.06	0.88 ± 0.07	<0.001
Fat% (%)	22.76 ± 4.77	25.50 ± 4.61	0.016	29.19 ± 6.16	32.41 ± 7.89	0.041
SBP (mm Hg)	127.13 ± 16.14	130.88 ± 9.38	0.124	121.78 ± 16.23	123.84 ± 16.58	0.399
DBP (mm Hg)	84.19 ± 9.38	84.44 ± 11.99	0.184	78.97 ± 9.53	77.22 ± 9.21	0.219
FPG (mmol/L)	4.83 (4.38–5.17)	7.33 (6.14–8.56)	<0.001	4.78 (4.50–5.17)	6.44 (5.58–7.22)	<0.001
2 h PG (mmol/L)	5.05 (4.00–6.00)	13.72 (10.97–16.14)	<0.001	5.39 (4.61–6.44)	12.67 (9.11–15.22)	<0.001
FINS (μU/ml)	17.63 (13.33–23.10)	19.48 (13.10–25.15)	0.048	17.74 (14.14–22.66)	20.13 (13.63–27.59)	0.04
2 h INS (μU/ml)	49.47 (30.32–79.73)	49.65 (31.57–75.34)	0.779	61.99 (42.18–91.93)	62.88 (43.35–106.00)	0.47
HOMA-IR	3.64 (2.72–5.03)	5.76 (3.78–8.91)	<0.001	3.76 (2.96–5.06)	5.77 (3.79–9.14)	<0.001
HbA1_C_ (%)	5.60 ± 0.46	7.11 ± 1.27	<0.001	5.57 ± 0.46	6.43 ± 1.05	<0.001
TC (mmol/L)	5.46 ± 1.00	5.59 ± 1.02	0.366	5.70 ± 1.08	5.81 ± 0.88	0.49
LDL-c (mmol/L)	2.32 ± 0.55	2.38 ± 0.59	0.456	2.49 ± 0.61	2.53 ± 0.47	0.662
HDL-c (mmol/L)	1.33 ± 0.33	1.24 ± 0.28	0.021	1.57 ± 0.36	1.45 ± 0.35	0.017
TG (mmol/L)	1.50 (1.05–2.21)	1.51 (1.16–2.38)	0.293	1.19 (0.87–1.64)	1.29 (1.04–1.96)	0.017
SFA (cm^2^)	123.00 (99.13–153.60)	130.95 (109.43–164.18)	0.081	182.95 (142.10–224.93)	170.80 (132.60–217.00)	0.162
VFA (cm^2^)	100.70 (53.72–135.70)	113.40 (70.27–142.95)	0.014	62.04 (43.48–86.88)	74.19 (50.73–95.23)	0.033
Total energy intake (kcal)	2249 .59 (1746.10–2581.79)	2348.54 (1808.94–2765.97)	0.546	1986.78 (1206.03–2266.45)	1709.98 (1155.23–2141.67)	0.672
Education level, *n* (%)						
Less than high school	29 (5.7)	7 (11.5)	0.38	91 (11.3)	11 (21.2)	0.068
High school	380 (73.9)	43 (70.5)		620 (77.0)	36 (69.2)	
More than high school	105 (20.4)	11 (18.0)		94 (11.7)	5 (9.6)	
Family income (RMB/person/month)						
Lower than 2000	13 (2.5)	2 (3.3)	0.702	55 (6.8)	3 (5.8)	0.567
2000–5000	261 (50.8)	34 (55.8)		543 (67.5)	36 (69.2)	
More than 5000	240 (46.7)	25 (40.9)		207 (25.7)	13 (25.0)	
Smoking (%)						
Current	206 (40.1)	21 (34.4)	0.385	44 (5.5)	2 (3.9)	0.415
Former	64 (12.4)	10 (16.4)		19 (2.3)	1 (1.9)	
Never	244 (47.5)	30 (49.2)		742 (92.2)	49 (94.2)	
Drinking (%)						
Drinker	305 (59.3)	35 (57.4)	0.759	95 (11.8)	4 (7.7)	0.302
No	209 (40.7)	26 (42.6)		710 (88.2)	48 (92.3)	
Physical activity (%)						
Light	364 (70.8)	43 (70.5)	0.97	608 (75.5)	42 (80.8)	0.525
Middle	181 (35.2)	24 (39.3)	0.588	162 (20.1)	8 (15.4)	0.267
Heavy	10 (1.9)	0 (0)	–	7 (0.9)	0 (0)	–

*BMI* body mass index, *WC* waist circumference, *WHR* waist-hip ratio, *Fat%* body fat percentage, *SBP* systolic blood pressure, *DBP* diastolic blood pressure, *FPG* fasting plasma glucose, *2* *h PG*: 2 h postprandial glucose, *FINS* fasting insulin, *2* *h INS*: 2 h insulin, *HOMA-IR* homeostatic model assessment of insulin resistance, *HbA1c* glycosylated hemoglobin A1c, *TC* total cholesterol, *LDL-c* low density lipoprotein-c, *HDL-c* high density lipoprotein-c, *TG* triglyceride, *SFA* subcutaneous fat area, *VFA* visceral fat area.

### Dietary patterns

We derived four major dietary patterns among 21 predefined food groups, and the associated factor loading scores with ≤−0.25 or ≥0.25 are shown in Table 2. The “vegetables-fruits” dietary pattern was represented by a high consumption of vegetables, beans, mushrooms, tubers, fruits, coarse cereals, seaweeds, wheat, nuts, and dairy products. The “rice–meat” pattern was characterized by high intake of red meat, white rice, poultry, animal organ meat, condiments, eggs, and beans. The “seafood-eggs” pattern included eggs, seafood, dairy products, nuts, fruits, and tea, and low intake of beverages. The “sweet-fast food” pattern was loaded heavily on fast foods, beverages, desserts, alcoholic beverages, and was inversely related on white rice and dairy products. Each dietary pattern showed a 12.33%, 12.29%, 8.41%, and 6.07% of the variation in food intake, respectively.

**Table 2:** Factor loadings for the four major dietary patterns derived from principal components analysis with orthogonal rotation.

Dietary patterns	Factor loading coefficient	Cumulative variance explained (%)
Food		
Vegetables–fruits pattern		
Vegetables	0.66	12.33
Beans	0.63	
Mushrooms	0.61	
Tubers	0.61	
Fruits	0.58	
Coarse cereal	0.53	
Seaweed	0.43	
Wheat	0.42	
Nuts	0.32	
Dairy products	0.27	
Rice–meat pattern		
Red Meat	0.76	24.62
White Rice	0.63	
Poultry	0.61	
Animal organ meat	0.48	
Condiments	0.35	
Eggs	0.28	
Beans	0.34	
Seafood–eggs pattern		
Eggs	0.67	33.03
Seafood	0.46	
Dairy products	0.35	
Nuts	0.31	
Fruits	0.31	
Tea	0.25	
Beverages	−0.25	
Sweet–fast food pattern		
Fast food	0.75	39.1
Beverages	0.7	
Dessert	0.63	
Alcoholic beverages	0.34	
White Rice	−0.25	
Dairy products	−0.29	

Factor loadings with absolute values ≥±0.25 were listed in the table among 21 food groups.

### Correlation of dietary pattern score with insulin resistance and fat accumulation

Table 3 showed the association between each dietary pattern scores with HOMA-IR. The vegetables–fruits pattern showed an inverse association (R = −0.250; *p* < 0.001 in male; and R = −0.152, *p* < 0.001 in female) with HOMA-IR. While sweet–fast food pattern presented a significant and positive association (R = 0.162, *p* = 0.002 in male, and R = 0.225, *p* < 0.001 in female) with HOMA-IR, when adjusted for age, smoking, drinking, education, economic income, total energy intake, physical activity, and BMI. In contrast, we found no significant association in the adjusted analysis for either the rice–meat pattern or the seafood–eggs pattern. Analyses of association between each dietary pattern score with VFA were also presented in Table 3. Vegetable-fruits pattern was negatively correlated with VFA in both males and females (R = −0.111, *p* = 0.029 in male, and R = −0.099 *p* = 0.017 in female). While sweet–fast food pattern was closely correlated with VFA in males but not in females (R = 0.200, *p* < 0.001 in male, and R = 0.012, *p* = 0.775 in female) after adjustment for age, smoking, drinking, education, economic income, total energy intake, and physical activity. Similarly, there was no association observed between the rice–meat pattern or the seafood–eggs pattern and VFA.

**Table 3:** Partial correlation analysis for the relationship between dietary pattern score and HOMA-IR and VFA.

Dietary pattern	Male		Female	
	r	*p* for trend	r	*p* for trend
HOMA-IR				
Vegetables–fruits pattern	−0.25	<0.001	−0.152	<0.001
Rice–meat pattern	−0.057	0.269	−0.047	0.26
Seafood–eggs pattern	−0.045	0.379	−0.022	0.606
Sweet–fast food	0.162	0.002	0.225	<0.001
VFA				
Vegetables–fruits pattern	−0.111	0.029	−0.099	0.017
Rice–meat pattern	−0.007	0.885	0.035	0.401
Seafood–eggs pattern	0.032	0.53	0.061	0.146
Sweet–fast food	0.2	<0.001	0.012	0.775

For DM, partial correlation analysis adjusted for age, smoking, drinking, education, economic income, total energy intake, physical activity and BMI. For central obesity, partial correlation analysis adjusted for age, smoking, drinking, education, economic income, total energy intake, and physical activity.

### The effect of dietary patterns on the risk of DM and central obesity

The risks of DM and central obesity were analyzed across the dietary pattern score tertiles (Table 4). Those participants in the highest tertile of vegetable-fruits pattern showed a significantly lower risk of DM in both males and females (OR: 0.30, 95% CI: 0.13–0.70 in male, and OR: 0.28, 95% CI: 0.11–0.72 in female), but lower risk of central obesity was observed only in males (OR: 0.50, 95% CI: 0.29–0.86 in male, OR: 1.48, 95% CI: 0.93–2.38 in female). Conversely, participants in the highest tertile of the sweet–fast food pattern demonstrated a higher risk of DM (OR: 2.58, 95% CI: 1.23–5.88 in male, and OR: 0.73, 95% CI: 0.29–1.85 in female), and central obesity (OR: 2.85,95% CI: 1.67–4.86 in male, and OR: 1.04, 95% CI: 0.66–1.64 in female) only in males. Neither the rice–meat pattern nor the seafood–eggs pattern showed significant association with DM or central obesity in both males and females.

**Table 4:** Associations between the dietary patterns and the risk of diabetes and central obesity.

	Male				Female			
Dietary pattern	Tertile 1	Tertile 2	Tertile 3	*p* for trend	Tertile 1	Tertile 2	Tertile 3	*p* for trend
*DM*								
Vegetables–fruits pattern								
No. of cases	191	185	199		285	287	285	
Crude-OR (95%)	1	0.54 (0.22–1.36)	0.28 (0.12–0.64)	0.005	1	0.75 (0.25–2.19)	0.26 (0.10–0.67)	0.003
Multivatiate adjusted-OR (95%)	1	0.53 (0.21–1.35)	0.30 (0.13–0.70)	0.012	1	0.80 (0.27–2.36)	0.28 (0.11–0.72)	0.005
Rice–meat pattern								
No. of cases	191	192	192		280	284	293	
Crude-OR (95%)	1	1.19 (0.53–2.68)	1.70 (0.79–3.67)	0.368	1	0.83 (0.32–2.15)	1.91 (0.85–4.30)	0.109
Multivatiate adjusted-OR (95%)	1	1.13 (0.49–2.60)	1.66 (0.77–3.62)	0.389	1	0.88 (0.34–2.28)	1.93 (0.86–2.28)	0.128
Seafood–eggs pattern								
No. of cases	192	192	191		284	288	285	
Crude-OR (95%)	1	1.70 (0.74–3.91)	0.83 (0.40–1.69)	0.189	1	1.09 (0.45–2.64)	1.44 (0.62–3.33)	0.664
Multivatiate adjusted-OR (95%)	1	2.31 (0.97–5.50)	1.02 (0.49–2.16)	0.087	1	1.06 (0.43–2.62)	1.43 (0.43–2.62)	0.673
Sweet–fast food								
No. of cases	189	194	192		275	297	285	
Crude-OR (95%)	1	1.62 (0.88–3.24)	2.65 (1.32–6.01)	0.026	1	1.33 (0.34–2.08)	0.84 (0.60–2.98)	0.546
Multivatiate adjusted-OR (95%)	1	1.53 (0.75–3.12)	2.58 (1.23–5.88)	0.044	1	1.26 (0.55–2.87)	0.73 (0.29–1.85)	0.464
*Central obesity*								
Vegetables–fruits pattern								
No. of cases	191	185	199		285	287	285	
Crude-OR (95%)	1	1.09 (0.66–1.81)	0.61 (0.37–1.01)	0.053	1	1.17 (0.74–1.86)	1.52 (0.97–2.38)	0.182
Multivatiate adjusted-OR (95%)	1	0.80 (0.44–1.36)	0.50 (0.29–0.86)	0.039	1	1.14 (0.71–1.82)	1.48 (0.93–2.38)	0.235
Rice–meat pattern								
No. of cases	191	192	192		280	284	293	
Crude-OR (95%)	1	1.00 (0.61–1.64)	1.12 (0.68–1.85)	0.87	1	1.12 (0.72–1.75)	1.00 (0.63–1.56)	0.835
Multivatiate adjusted-OR (95%)	1	1.18 (0.69–2.03)	1.49 (0.83–2.68)	0.405	1	1.21 (0.72–2.03)	1.24 (0.77–1.99)	0.65
Seafood–eggs pattern								
No. of cases	192	192	191		284	288	285	
Crude-OR (95%)	1	1.12 (0.68–1.85)	1.02 (0.62–1.68)	0.887	1	0.85 (0.54–1.32)	0.82 (0.53–1.28)	0.638
Multivatiate adjusted-OR (95%)	1	1.15 (0.69–1.94)	1.09 (0.66–1.82)	0.864	1	0.85 (0.54–1.34)	0.82 (0.51–1.31)	0.673
Sweet–fast food								
No. of cases	189	194	192		275	297	285	
Crude-OR (95%)	1	1.89 (1.13–3.16)	2.62 (1.56–4.40)	0.001	1	1.17 (0.75–1.84)	0.97 (0.62–1.52)	0.674
Multivatiate adjusted-OR (95%)	1	1.93 (1.14–3.29)	2.85 (1.67–4.86)	<0.001	1	1.25 (0.79–1.98)	1.04 (0.66–1.64)	0.582

For DM, multivatiate model adjusted for age, smoking, drinking, education, economic income, total energy intake, physical activity, and BMI. For central obesity, multivatiate model adjusted for age, smoking, drinking, education, economic income, total energy intake, and physical activity.

## Discussion

In the present study, we investigated the association of dietary patterns with the risk of DM and central obesity in the middle- aged Chinese population. We extracted four major dietary patterns using factor analysis: vegetables–fruits food pattern, rice–meat pattern, seafood–eggs pattern, and sweet–fast food pattern. Vegetables–fruits food pattern and sweet–fast food pattern were associated with DM and central obesity; however, neither rice–meat food pattern nor seafood–eggs pattern were associated with DM or central obesity.

The vegetables–fruits pattern is generally considered as a healthy pattern. Previous studies have reported that the fruits and vegetables, particularly intake of high green leafy vegetables, contribute to a reduced risk of T2DM and obesity^25–28^. The protective effects of this pattern may be attributable to the healthy constituents, which contain abundant dietary fiber but a low glycemic index^29,30^. In addition, vegetables and fruits were considered as potent antioxidants^31^, and some studies have supported this hypothesis^32,33^. Overall, these findings suggested that vegetables and fruits provided benefits for decreasing the prevalence of DM and central obesity among Chinese population.

Sweet–fast food pattern characterized by high consumption of desserts, fast foods, and beverages was significantly associated with a higher prevalence of DM and central obesity. The results are consistent with the existing studies. Fast food tends to be energy-dense, high in glycemic load, poor in fiber and low in micronutrients. Positive association between the fast food and obesity and T2DM have been observed in several previous studies^34–36^. In addition, it was assessed that people who consumed more sweetened beverages and desserts showed a higher risk of developing central obesity, IR and DM^28,37–39^, because of high glycemic index and glycemic load. However, the study participants were restricted to the middle- aged Chinese population, who rarely consume fast foods, dessert, and drinks, and this may partly affect the outcomes.

In our study, we found no association of rice–meat pattern or seafood–eggs pattern with DM or central obesity. Red meat and white rice were the major components of rice–meat pattern. Red meat, which contains huge amount of saturated fat and cholesterol, is considered to be an energy-dense food and excess consumption may contribute to a surplus intake of energy, which may in turn increase the risk of T2DM and obesity^40,41^. However, few studies showed no relationship between red meat and T2DM or obesity^22^. This may be due to high content of zinc in red meat. Zinc content is highest in animal-source foods, and low in refined cereals and vegetables^42^. Zinc deficiency may aggravate IR in non-insulin dependent DM over a 6 year follow-up study^43^. According to a previous systematic review conducted among the Asian populations, rice which had a high glycemic index was positively associated with the risk of developing T2DM and weight gain^30,44,45^. However, studies aiming to investigate the association between rice intake and propensity to develop obesity are still inconclusive^45–47^. Furthermore, study participants with obesity and DM risk might have been advised to limit their consumption of carbohydrate and fat, which would influence the results.

Egg and seafood had a high factor loading in the seafood–eggs pattern. Egg is a nutrient-dense food containing high-quality protein and amount of beneficial nutrients. However, dietary cholesterol from eggs can also lead to a modest increase in blood liquids. The results of previous studies focused on egg consumption and IR and T2DM were controversial. A meta-analysis by Djousse et al. showed no significant effect of egg consumption on T2DM^48^. While the China National Nutrition Survey reported that egg consumption more than one time per day increased the T2DM occurrence in females and that TG and TC were significantly higher in females taking two or more eggs per week^49^. On the other hand, a study conducted in Korean reported that consumption of more than one egg per day could decrease the risk of metabolic syndrome (MetS)^50^. Different results on the association were likely due to differences in dietary patterns across countries. Data from previous studies in relation to the intake of fish with DM risk also are inconclusive. Ecological studies in several countries have shown protective effects of fish intake against the body weight gain and the development of DM^51,52^. However, the beneficial effects were not evident in other studies^53,54^, which was consistent with our study. A recent systematic review and meta-analysis of fish consumption and risk of T2DM suggested heterogeneity between geographical regions in the observed association^55^. The different associations of fish consumption with DM and obesity in different populations could be partly explained by diverse fish preparation methods, different type and amount of cooking fat used, and various kind of condiments.

In the present study, we found that the effect of food patterns might be greater in males than in females for DM and central obesity. Difference between the genders in the physiological response to different food patterns is uncertain, one plausible explanation is the different anatomy and physiology between males and females. For example, female may convert more α-linoleic acid into docosahexanoic acid than male do. In addition, the study participants are middle-aged Chinese population, and at times few female participants are involved in peri- and postmenopausal period. Previous studies proved that menopause-related loss of estrogen and increased androgens appear to be key hormonal changes that is associated with the development of MetS, which were independent of aging and other standard cardiovascular disease risk factors^56^. The study power of stratified analysis was limited due to the number of participants. Additional large population are needed in the future.

There were several potential limitations of this study that need to be highlighted. Firstly, because our study is a cross-sectional design study, no cause-and-effect relationships between dietary patterns and the risk of DM and central obesity may be established. Secondly, several arbitrary and subjective decisions in the use of factor analysis should be taken into consideration. Thirdly, even though we have adjusted for confounding factors that are related to the risk of DM and obesity, we cannot exclude the influence of uncontrolled confounders on our observed results. Finally, the study sample was composed of the middle-aged Chinese population in Hangzhou, Zhejiang Province, eastern China, and these results cannot be generalized to the entire Chinese population. In conclusion, this study has identified that vegetables–fruits food pattern was significantly associated with a lower risk of DM and central obesity, while the reverse was apparent for sweet–fast food pattern among Chinese population aged 40–65 years. Nevertheless, further prospective studies are needed to understand the causal relationships between dietary patterns and the risk of central obesity and DM.

## References

[1] National Diabetes Research Group. (1981). A mass survey of diabetes mellitus in a population of 300,000 in 14 provinces and municipalities in China (in Chinese). Zhonghua Nei Ke Za Zhi.

[2] Pan XR, Yang WY, Li GW, Liu J (1997). Prevalence of diabetes and its risk factors in China, 1994. National Diabetes Prevention and Control Cooperative Group. Diabetes Care.

[3] Yang SH, Dou KF, Song WJ (2010). Prevalence of diabetes among men and women in China. N. Engl. J. Med..

[4] Xu Y (2013). Prevalence and control of diabetes in Chinese adults. JAMA.

[5] Lu C, Sun W (2014). Prevalence of diabetes in Chinese adults. JAMA.

[6] Wang L (2017). Prevalence and ethnic pattern of diabetes and prediabetes in China in 2013. JAMA.

[7] Yin M, Augustin B, Shu C, Qin T, Yin P (2016). Probit models to investigate prevalence of total diagnosed and undiagnosed diabetes among aged 45 years or older adults in China. PloS ONE.

[8] Hossain P, Kawar B, El Nahas M (2007). Obesity and diabetes in the developing world-a growing challenge. N. Engl. J. Med..

[9] Gur EB (2014). Ultrasonographic visceral fat thickness in the first trimester can predict metabolic syndrome and gestational diabetes mellitus. Endocrine.

[10] Kahn SE (2001). Obesity, body fat distribution, insulin sensitivity and Islet beta-cell function as explanations for metabolic diversity. J. Nutr..

[11] Alberti KG, Zimmet P, Shaw J (2006). Metabolic syndrome-a new world-wide definition. A Consensus Statement from the International Diabetes Federation. Diabet. Med. J. Br. Diabet. Assoc..

[12] Rodriguez-Monforte M, Flores-Mateo G, Sanchez E (2015). Dietary patterns and CVD: a systematic review and meta-analysis of observational studies. Br. J. Nutr..

[13] Schrijvers, J. K., McNaughton, S. A., Beck, K. L. & Kruger, R. Exploring the dietary patterns of young new Zealand women and associations with BMI and body frat. *Nutrients*10.3390/nu8080450 (2016). 10.3390/nu8080450 PMC4997365 27472358

[14] Spence M, McKinley MC, Hunter SJ (2010). Session 4: CVD, diabetes and cancer: diet, insulin resistance and diabetes: the right (pro)portions. Proc. Nutr. Soc..

[15] Tremblay A, Gilbert JA (2009). Milk products, insulin resistance syndrome and type 2 diabetes. J. Am. Coll. Nutr..

[16] Gunji T (2011). Alcohol consumption is inversely correlated with insulin resistance, independent of metabolic syndrome factors and fatty liver diseases. J. Clin. Gastroenterol..

[17] Flanagan DE (2000). Alcohol consumption and insulin resistance in young adults. Eur. J. Clin. Invest..

[18] He Y (2009). Dietary patterns and glucose tolerance abnormalities in Chinese adults. Diabetes Care.

[19] Villegas R (2010). Dietary patterns are associated with lower incidence of type 2 diabetes in middle-aged women: the Shanghai Women’s Health Study. Int. J. Epidemiol..

[20] Zuo H (2013). Dietary patterns are associated with insulin resistance in Chinese adults without known diabetes. Br. J. Nutr..

[21] Jia Q (2015). Dietary patterns are associated with prevalence of fatty liver disease in adults. Eur. J. Clin. Nutr..

[22] Zhang M (2015). Associations between dietary patterns and impaired fasting glucose in Chinese men: a cross-sectional study. Nutrients.

[23] Matthews DR (1985). Homeostasis model assessment: insulin resistance and beta-cell function from fasting plasma glucose and insulin concentrations in man. Diabetologia.

[24] Bao Y (2008). Optimal waist circumference cutoffs for abdominal obesity in Chinese. Atherosclerosis.

[25] Li M, Fan Y, Zhang X, Hou W, Tang Z (2014). Fruit and vegetable intake and risk of type 2 diabetes mellitus: meta-analysis of prospective cohort studies. BMJ Open.

[26] Schwingshackl L (2017). Food groups and risk of type 2 diabetes mellitus: a systematic review and meta-analysis of prospective studies. Eur. J. Epidemiol..

[27] Schwingshackl L (2015). Fruit and vegetable consumption and changes in anthropometric variables in adult populations: a systematic review and meta-analysis of prospective cohort studies. PLoS ONE.

[28] van Eekelen E (2019). Sweet snacks are positively and fruits and vegetables are negatively associated with visceral or liver fat content in middle-aged men and women. J. Nutr..

[29] Murakami K (2007). Dietary fiber intake, dietary glycemic index and load, and body mass index: a cross-sectional study of 3931 Japanese women aged 18-20 years. Eur. J. Clin. Nutr..

[30] Sugiyama M, Tang AC, Wakaki Y, Koyama W (2003). Glycemic index of single and mixed meal foods among common Japanese foods with white rice as a reference food. Eur. J. Clin. Nutr..

[31] Hamer M, Chida Y (2007). Intake of fruit, vegetables, and antioxidants and risk of type 2 diabetes: systematic review and meta-analysis. J. Hypertension.

[32] Salonen JT (1995). Increased risk of non-insulin dependent diabetes mellitus at low plasma vitamin E concentrations: a four year follow up study in men. BMJ.

[33] Reunanen A, Knekt P, Aaran RK, Aromaa A (1998). Serum antioxidants and risk of non-insulin dependent diabetes mellitus. Eur. J. Clin. Nutr..

[34] Li M, Dibley MJ, Sibbritt DW, Yan H (2010). Dietary habits and overweight/obesity in adolescents in Xi’an City, China. Asia Pac. J. Clin. Nutr..

[35] Shu L (2015). Association between dietary patterns and the indicators of obesity among Chinese: a cross-sectional study. Nutrients.

[36] Mazidi, M. & Speakman, J. R. Association of fast-food and full-service restaurant densities with mortality from cardiovascular disease and stroke, and the prevalence of diabetes mellitus. *J. Am. Heart Assoc.*10.1161/JAHA.117.007651 (2018). 10.1161/JAHA.117.007651 PMC6015353 29802148

[37] Ma J (2016). Sugar-sweetened beverage but not diet soda consumption is positively associated with progression of insulin resistance and prediabetes. J. Nutr..

[38] O’Connor L (2018). Intakes and sources of dietary sugars and their association with metabolic and inflammatory markers. Clin. Nutr..

[39] Karatzi K (2014). Dietary patterns and breakfast consumption in relation to insulin resistance in children. The Healthy Growth Study. Public Health Nutr..

[40] Vergnaud AC (2010). Meat consumption and prospective weight change in participants of the EPIC-PANACEA study. Am. J. Clin. Nutr..

[41] Aune D, Ursin G, Veierod MB (2009). Meat consumption and the risk of type 2 diabetes: a systematic review and meta-analysis of cohort studies. Diabetologia.

[42] Brown KH (2004). International Zinc Nutrition Consultative Group (IZiNCG) technical document #1. Assessment of the risk of zinc deficiency in populations and options for its control. Food Nutr. Bull..

[43] Vashum KP (2013). Is dietary zinc protective for type 2 diabetes? Results from the Australian longitudinal study on women’s health. BMC Endocr. Disord..

[44] Lambert E (2011). Change in sympathetic nerve firing pattern associated with dietary weight loss in the metabolic syndrome. Front. Physiol..

[45] Shi Z, Taylor AW, Hu G, Gill T, Wittert GA (2012). Rice intake, weight change and risk of the metabolic syndrome development among Chinese adults: the Jiangsu Nutrition Study (JIN). Asia Pac. J. Clin. Nutr..

[46] Kim J, Jo I, Joung H (2012). A rice-based traditional dietary pattern is associated with obesity in Korean adults. J. Acad. Nutr. Dietetics.

[47] Kolahdouzan M (2013). The association between dietary intake of white rice and central obesity in obese adults. ARYA Atheroscler..

[48] Djousse L, Khawaja OA, Gaziano JM (2016). Egg consumption and risk of type 2 diabetes: a meta-analysis of prospective studies. Am. J. Clin. Nutr..

[49] Shi Z, Yuan B, Zhang C, Zhou M, Holmboe-Ottesen G (2011). Egg consumption and the risk of diabetes in adults, Jiangsu, China. Nutrition.

[50] Shin, S. et al. Egg consumption and risk of metabolic syndrome in Korean adults: results from the Health Examinees Study. *Nutrients*10.3390/nu9070687 (2017). 10.3390/nu9070687 PMC5537802 28671590

[51] Feskens EJ (1995). Dietary factors determining diabetes and impaired glucose tolerance. A 20-year follow-up of the Finnish and Dutch cohorts of the Seven Countries Study. Diabetes Care.

[52] Nkondjock A, Receveur O (2003). Fish-seafood consumption, obesity, and risk of type 2 diabetes: an ecological study. Diabetes Metab..

[53] Xun PC, He K (2012). Fish consumption and incidence of diabetes meta-analysis of data from 438,000 individuals in 12 independent prospective cohorts with an average 11-year follow-up. Diabetes Care.

[54] Ramadeen A (2012). N-3 polyunsaturated fatty acid supplementation does not reduce vulnerability to atrial fibrillation in remodeling atria. Heart Rhythm. Off. J. Heart Rhythm. Soc..

[55] Wallin A (2012). Fish consumption, dietary long-chain n-3 fatty acids, and risk of type 2 diabetes: systematic review and meta-analysis of prospective studies. Diabetes Care.

[56] Janssen I, Powell LH, Crawford S, Lasley B, Sutton-Tyrrell K (2008). Menopause and the metabolic syndrome: the Study of Women’s Health Across the Nation. Arch. Intern. Med..

